# Characterization of Inkjet-Printed Digital Microfluidics Devices

**DOI:** 10.3390/s21093064

**Published:** 2021-04-28

**Authors:** Shiyu Chen, Zhidong He, Suhwan Choi, Igor V. Novosselov

**Affiliations:** Department of Mechanical Engineering, University of Washington, Seattle, WA 98195, USA; shiyuc3@uw.edu (S.C.); zdhe21@uw.edu (Z.H.); choish37@gmail.com (S.C.)

**Keywords:** digital microfluidics, PMMA, Kapton, Ag ink, inkjet printing

## Abstract

Digital microfluidics (DMF) devices enable precise manipulation of small liquid volumes in point-of-care testing. A printed circuit board (PCB) substrate is commonly utilized to build DMF devices. However, inkjet printing can be used to fabricate DMF circuits, providing a less expensive alternative to PCB-based DMF designs while enabling more rapid design iteration cycles. We demonstrate the cleanroom-free fabrication process of a low-cost inkjet-printed DMF circuit. We compare Kapton and polymethyl methacrylate (PMMA) as dielectric coatings by measuring the minimal droplet actuation voltage for a range of actuation frequencies. A minimum actuation voltage of 5.6 V was required for droplet movement with the PMMA layer thickness of 0.2 μm and a hydrophobic layer of 0.17 μm. Significant issues with PMMA dielectric breakdown were observed at actuation voltages above 10 V. In comparison, devices that utilized Kapton were found to be more robust, even at an actuation voltage up to 100 V.

## 1. Introduction

Digital microfluidics (DMF) is an approach for manipulating small liquid volumes using electrostatic force; it allows for the precise control of individual droplet movement in microliter to nanoliter volumes in an array of electrodes [1]. DMF can perform droplet operations such as dispensing, mixing, splitting, and merging without the external sources like pumps and valves that channel-based microfluidics require [2]. Complex multistep operations (e.g., washing, solvent exchanges, and extractions) can also be automated utilizing generic device geometry. Typically, there are two standard configurations of DMF devices—one-plate and two-plate devices. In the one-plate format, droplets are manipulated on a single substrate bearing both actuation and ground electrodes. In the two-plate format, droplets are sandwiched between a counter-electrode affixed on the top plate and a bottom plate bearing an array of insulated driving electrodes. Two-plate devices can implement more operations, most notably dispensing and splitting [3]. Two-plate DMF devices can be operated in air or silicone oil [4,5]. Although the operation procedures in an open system are more straightforward, lower actuation voltages can be used due to a reduction in surface tension in an oil-immersed system; the evaporation of droplets is also eliminated [3].

The fabrication of DMF devices is challenging and is often complicated by the need for a cleanroom. A common way to manufacture DMF chips is to utilize printed circuit boards (PCBs) because of their relatively low production cost and multilayer format, enabling complex electrode and wiring designs [3,4]. However, the deposition of dielectric and hydrophobic layers onto a PCB-DMF device is tedious and time-consuming; thus, the preproduction design cycle of PCB-DMF can be rather long. Inkjet printing and other deposition methods have been utilized for microfluidic devices due to their low cost. They are suitable for mass production, shortening the fabrication time and the design cycle [5,6]. Novel printing and coating methods have recently been reported for wearable devices, e.g., [7,8,9,10]. The deposition of conductive metals (e.g., Au, Ag, Cu) [2] and carbon-based inks such as carbon nanotubes [3] and graphene [4] on flexible substrates has been previously reported. Silver inks are particularly attractive for fabrication in the flexible circuit, as silver has the highest electric conductivity among metals. Ag has excellent chemical stability, relatively low cost (compared to Au), and its oxides are also conductive. Fernandes et al. reported the effect of Ag ink formulations on inkjet printability, related to properties such as resistivity, viscosity, surface tension, and adhesion [5].

In DMF devices, electrodes are first covered with a dielectric layer; then, a hydrophobic coating is applied to ensure smooth droplet movement [11]. The effects of coating thickness, deposition uniformity, high initial contact angle, and low contact angle hysteresis should be considered when choosing the hydrophobic material [12]. The common materials include Teflon, Parafilms, FluoroPel, Cytop, and polydimethylsiloxane (PDMS) [13]. Other commercially available dielectric and hydrophobic coatings can be used; their parametrization is beyond the scope of this paper. These materials’ thicknesses vary from 50 μm to 100 μm, and the actuation voltages (*φ*) may vary from 50 V to 800 V [14,15,16]. The actuation voltages are primarily affected by the dielectric layer’s thickness, and materials may fail when the applied voltage exceeds a certain limit [17]. Polymeric and inorganic materials are widely used in the manufacturing of dielectric layers. Parylene-C is often used as an insulating coating in DMF because of its relatively high dielectric constant and lower voltage, which changes the contact angle [17]. Silicon dioxide (SiO_2_) can serve as an inorganic insulator due to its high dielectric constant and strength [18]. Recently, PMMA and Kapton tape have been used as dielectric layers in DMF. The main advantages are their low cost, simple manufacturing process, and low thickness, which can translate to lower droplet actuation voltage. Parametric characterization of these coatings is required to advance the fabrication of low-cost DMF devices. Other commercially available dielectric and hydrophobic coatings can be used; their parametrization is beyond the scope of this paper. The stability of these materials under repeated exposure to high electric fields also requires investigation.

This paper presents a methodology for the fabrication of a DMF circuit using an inkjet printer, followed by coating by dielectric and hydrophobic materials. The cartridge design is amenable to cleanroom-free low-cost fabrication. A simple five-electrode DMF cartridge is used to demonstrate droplet manipulation. Two dielectrics, Kapton and PMMA, were tested in terms of droplet actuation frequency and minimum actuation voltage. All fabrication steps were performed outside the cleanroom, enabling rapid, low-cost DMF chip fabrication.

## 2. Materials and Methods

### 2.1. Material and Equipment

The inkjet-printed DMF circuit was fabricated using a Fujifilm Dimatix Material Printer (DMP) by depositing silver nanoparticle ink (Ag ink) onto polyethylene terephthalate (PET) film. The thickness of the PMMA dielectric layer and the hydrophobic layer on Kapton^®^ tape was measured with an optical profilometer (Olympus OLS41) with 100× magnification.

The power source consisted of a function generator (BK Precision 4052, Yorda Linda, CA, USA) and a high voltage (HV) amplifier (Trek PZ700, Advanced Energy, Dever, CO, USA). A DAQpad-6229 BNC digital I/O board from National Instruments Co. (NI, Fort Collins, CO, USA) was used to energize the relays (AQW614EH, Panasonic, Osaka, Japan) linked to the individual electrode. The system was controlled using a custom LABVIEW code (NI, Fort Collins, CO, USA). The top covers were glass slides with indium tin oxide (ITO)-coated PET film with a surface resistivity of 60 Ω/sq (Sigma Aldrich, St Louis, MO, USA), which surfed as a counter electrode. The substrates were cleaned using oxygen plasma (Barrel Etch AutoGlow, Tempe, AZ, USA) for 1 min to facilitate good coating layer adhesion. A spin coater was used to deposit the dielectric and hydrophobic layers. Dielectric materials included Kapton or 0.4%, 1.5%, and 4% by weight of PMMA in anisole (Sigma Aldrich, St Louis, MO, USA). A FluoroPel PFC1101V (Cytonix, Beltsville, MD, USA) was used to apply the hydrophobic layer. Silicone oil of 1 CST (Sigma Aldrich, St Louis, MO, USA) was applied as filler media in the cartridge.

### 2.2. Device Fabrication

**Figure 1:**
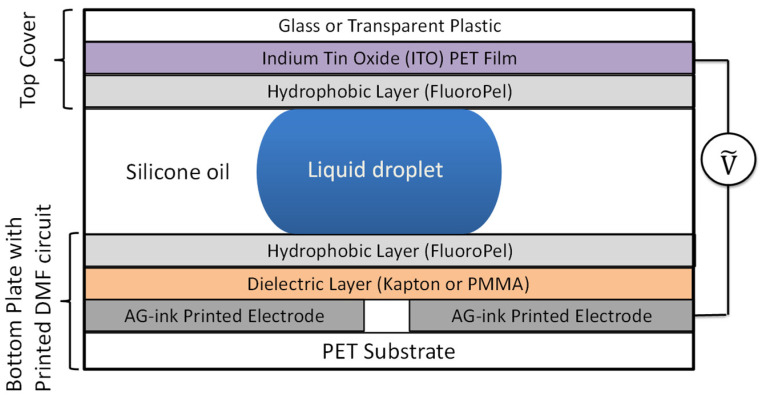
Conceptual diagram showing the assembly layers of the DMF cartridge with Ag-ink-printed electrodes. The schematic is not drawn to scale.

The DMF chip fabrication protocol consisted of printing DMF electrodes, applying coatings to the top and bottom plates and assembling the cartridge. Figure 1 shows the conceptual diagram of the assembled device.

The DMF circuits were printed using a DMP-2800 with silver nanoparticle ink (Ag ink) (Sigma Aldrich, St Louis, MO, USA). The DMP settings were 22-volt firing voltage, 700-micrometer print height and 25-micrometer drop spacing; a single printing nozzle was used. The Ag ink was a dispersion of Ag nanoparticles (<150 nm) in an organic solvent (ethanol and ethanediol) with a 20 wt % concentration. No issues such as flaking, cracking, or separation between the layers were observed during printing on PET substrates. Particle adhesion to the surface is driven by surface energies. Preferably, the surface energies should not be very low, as this may lead to poor quality prints. For example, van Osch et al. reported the formation of bulges in conducting tracks during the printing of Teflon substrates with lower surface energy (*γ* = 18 mJ/m^2^), whereas printing onto the PET substrate (*γ* ~40 mN/m) produced good quality prints [19].

**Figure 2:**
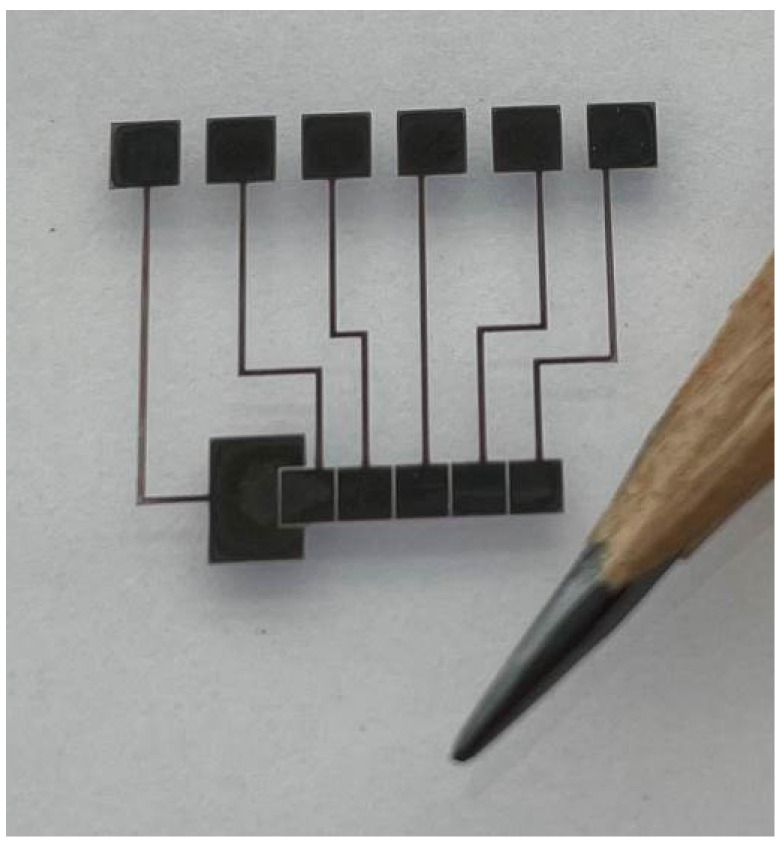
Photograph of Ag-ink-printed DMF circuit on a PET substrate.

As suggested by a previous report, to achieve low electrical resistivity of Ag ink [20], the printed samples were ultraviolet (UV) cured for 480 s. Figure 2 shows the printed circuit on the PET substrate; the design consists of one larger electrode for droplet introduction and five smaller electrodes, 1.5 mm × 1.5 mm, for droplet transport. The electrodes are separated by a 100-μm gap. Each electrode is connected to a 1.8 mm × 1.8 mm pad for interface with the power supply via a 100-μm-wide lead. The quality of prints was visually inspected using an optical microscope to ensure proper spacing between the electrodes and the tracers’ thickness.

The bottom plate consists of a glass coverslip, a PET substrate with the printed circuit, a dielectric layer, and a hydrophobic layer. The top cover consists of a glass slide, ITO PET film, and a hydrophobic layer. The diameter of the holes is compatible with a standard pipette tip.

Surface defects such as dust or scratches can seriously affect the droplet movement; therefore, a plasma cleaner was used to clean the PET substrate with the printed circuit. The PET substrate was attached to the glass coverslip with double-sided adhesive tape.

Before applying the PMMA layer, the contact pads were masked using adhesive tape to prevent PMMA deposition on the electrical contacts. For bottom plate fabrication, 100 µL of PMMA reagent was used. The spin coater speed was set to 500 rpm for 10 s, followed by 1500 rpm for 30 s. After drying at room temperature for 20 min, 100 µL of Fluoropel solution was applied, and the spin coater was set at 500 rpm for 10 s, followed by 1750 rpm for 30 s. The top cover ports were drilled from the PET side to avoid breaking and chipping the glass. Before assembly, all parts were dusted with clean compressed air.

**Figure 3:**
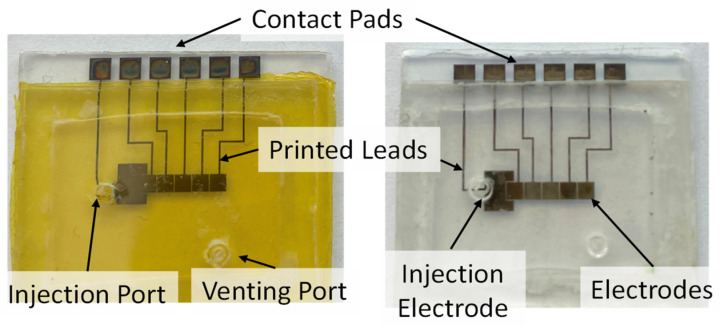
Left—assembled two-plate DMF cartridge with Kapton tape dielectric layer. Right—assembled two-plate DMF cartridge with PMMA dielectric layer.

The precut silicon gasket was fixed to the bottom plate with UV-sensitive adhesive. The adhesive was cured in a UV box for 10 min. The corners between the gasket and bottom plate were touched with the hydrophobic solution to fill any gaps. The top cover was glued to the bottom plate, and the entire assembly was UV cured for 30 min. Before the droplet test, the cartridge was filled with silicone oil to reduce the surface tension between the droplet and the components; then, a 1 -μL droplet was injected through the injection port; see Figure 3.

### 2.3. Device Operation

**Figure 4:**
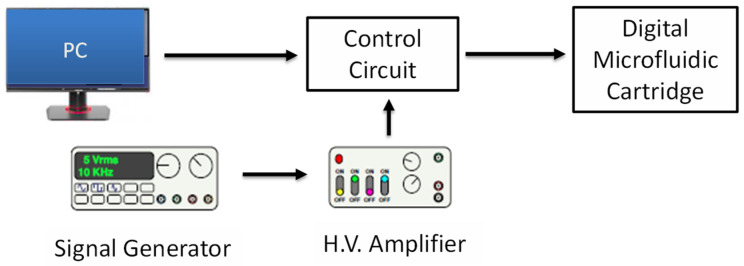
Diagram of DMF system. The function generator signal is amplified in the high-voltage amplifier and passed through the control circuit to a specific electrode.

To operate the DMF circuit, the actuation signal was applied to the electrodes. The voltage was varied to determine the minimum operating potential for each droplet movement frequency. Figure 4 shows a diagram of the experimental setup. The control circuit was controlled using a LABVIEW program to pass the actuation voltage to the specific electrodes. We evaluated droplet movement frequency (*f*), defined by the number of times a droplet moved back and forth between two electrodes per second. The frequency was set to *f* = 0.2 Hz, 1 Hz, 2 Hz, and 5 Hz by the computer. For each frequency, a range of voltages was applied; *φ* = 10–300 V. The conditions were chosen to examine the stability of the coating and droplet actuation.

## 3. Results and Discussions

### 3.1. Coating Thickness Evaluation

**Figure 5:**
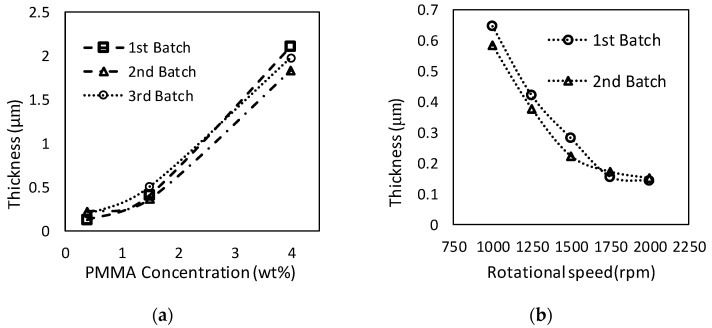
(**a**) Thickness of the PMMA layer as a function of PMMA concentration in anisole (0.4%, 1.5%, and 4%); the coater rotational speed was set to 1750 rpm. (**b**) Thickness of Fluoropel layer for spin coater settings of 1000 rpm, 1250, 1500 rpm, 1750 rpm, and 2000 rpm on Kapton.

Coating thickness was evaluated as a function of deposition parameters. Figure 5a shows the PMMA layer’s thickness plotted against the PMMA concentration in the anisole solution. The PET substrate thickness was measured before the coating procedure and again after coating. To evaluate the repeatability of the procedure, three batches for each concentration were prepared. The thickness of PMMA samples ranged from 0.17 ± 0.05 μm to 1.97 ± 0.14 μm. The thickness increased with the weight percentage of the PMMA in the solution. The required actuation voltage was lower for thinner dielectric layers; however, the thinner dielectric layer led to dielectric breakdown, limiting the actuation voltage range.

The effect of rotational speed on FluoroPel deposition thickness was studied; a thinner layer is desired in order to reduce the cost of reagents. Figure 5b shows the thickness of hydrophobic layers coated on Kapton tape with five different revolution speeds. The thickness of Kapton tape was measured before and after the coating to calculate the deposition thickness. The Fluoropel layer thickness decreased as the rotational speeds increased. The hydrophobic layer’s target thickness was ~0.2 μm, so speeds >1500 rpm were acceptable. Considering that similar thicknesses were achieved for 1750 rpm and 2000 rpm, 1750 rpm was chosen for the cartridge fabrication.

### 3.2. Droplet Movement Evaluation

Both PMMA and Kapton cartridges were tested. In each experiment, a water droplet (1 μL) colored using food coloring was injected into the cartridge, and the voltage was applied to the DFM circuit. A video demonstrating droplet movement is presented in the Supplemental Information.

**Figure 6:**
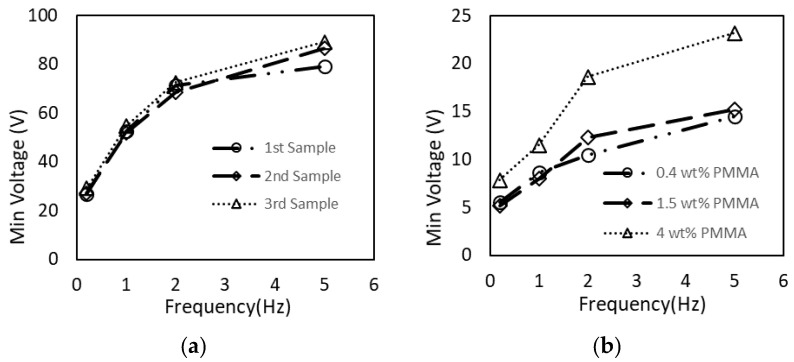
Minimal droplet actuation voltages for *f* = 0.2 Hz, 1 Hz, 2 Hz, and 5 Hz. (**a**) Three identical Kapton DMF devices were tested; (**b**) PMMA DMF for three different recipes, i.e., different dielectric thicknesses.

In the Kapton DMF cartridge experiments, three different chips were tested at four different frequencies. Figure 6a shows that all three samples had similar characteristics, i.e., minimal actuation voltages vs. actuation frequency were nearly identical for all cartridges. As the input frequency increases, the system required higher voltages to oscillate the droplet. For *f* = 0.2 Hz, the minimum voltage was 27 V; for *f* = 5 Hz, the minimal actuation voltage was 85 V. The increase in the required voltage at high frequencies points to the system’s hysteresis, i.e., the system needs to re-equilibrate after the initial droplet movement and before the movement in the opposite direction can occur. At the higher frequency, higher Coulombic forces are required to overcome the low angle hysteresis, inertia, surface tension, and other physiochemical interactions associated with the liquid–surface–charge interaction. Detailed analysis of this transient regime requires further investigation, and is beyond the scope of this manuscript.

PMMA-coated DMF cartridges with three different PMMA thicknesses were tested (0.17 μm, 0.43 μm, and 1.97 μm). Figure 6b shows the minimum voltage required to manipulate the droplet as a function of actuation frequency. As expected, the devices prepared with lower PMMA concentrations (thinner dielectric layers) required lower actuation voltages. For 0.2 Hz, the PMMA-coated samples only needed around 6 V to initiate the droplet transport. The maximum required voltage was ~20 V at 5 Hz and a 2-μm PMMA thickness (4% concentration). However, dielectric breakdown was observed for the PMMA-coated chip. At *φ* > 10 V, bubbles formed around the energized electrode. The bubbles completely filled up the space around the droplet, blocking it from moving at *φ* > 20 V. To determine the nature of the obstruction, the liquid was evacuated, and the surface was examined under an optical microscope. No damage to the coating was observed. Thus, we concluded that the bubbles were formed due to the electrolysis of water when the droplet was exposed to the high electric field. In the case of Kapton, the increased dielectric layer thickness reduced the propensity for electrolysis, and it is likely that this effect may be observed at higher voltages and vice versa; thicker PMMA insulation is likely to reduce bubble formation. We did not observe the delamination of PMMA or Kapton in our experiments after their exposure to electric fields.

The PMMA layer was significantly thinner (0.2–2 μm) compared to the Kapton layer (~30 μm). The electrical field strength for the Kapton DMF device at the droplet interface was lower for the same actuation voltage. Thus, to reach the threshold for droplet movement, a higher actuation voltage was required.

## 4. Conclusions

This study presents proof-of-concept experiments for inkjet-printed DMF circuits. We report the fabrication method for a simple DMF chip. In the fabrication of this chip, we employed two different dielectric layers: Kapton (~30 μm) and PMMA (0.2–2 μm). FluoroPel coating was used as a hydrophobic layer. Low dielectric thicknesses of the PMMA coating enabled low thresholds for droplet manipulation. In the case of the 0.2-μm PMMA layer, followed by 0.17 μm FlouroPel, the actuation threshold was ~ 5.6 V. For the Kapton DMF device, the minimum actuation voltage was *φ* = 26.8 V. Both coatings showed low actuation voltages than those typically used for DMF devices [14,15,16]. In terms of durability, the PMMA-coated circuit showed the formation of gas bubbles at the energized electrode due to the electrolysis of water, which blocked droplet movement. The Kapton-coated printed circuit was more robust, making it more suitable for DMF fabrication in our case. Specific attention should be paid to the dielectric layer’s effect on the performance of DMF devices in future designs. Although this study provides a demonstration of droplet actuation, scaling the circuit’s complexity and implementing the high-volume fabrication of DMF devices provide additional challenges.

## Data Availability

All data are presented in the manuscript and in the Supplemental Information.

## Supplementary Materials

The following are available online at https://www.mdpi.com/article/10.3390/s21093064/s1, Video S1: Demonstration of droplet movements in the PMMA-coated DMF device.
